# A Study on the Mechanism of Acetyl Tributyl Citrate-Induced Infertility Toxicity and the Protective Action of Icariin Based on Network Toxicology, Network Pharmacology, Molecular-Docking Technology and Molecular Dynamics Simulation

**DOI:** 10.3390/ijms27062918

**Published:** 2026-03-23

**Authors:** Xiaowei Sun, Peng Chen, Yuxing Han, Yuqing Du, Siyu Sun, Jin Miu, Xueying Li, Shaobo Liu, Chunlei Wan

**Affiliations:** 1 College of Life Science and Technology, Mudanjiang Normal University, Mudanjiang 157011, China; swxsxw@126.com (X.S.); cp2864882974@163.com (P.C.); hyx20010907@163.com (Y.H.); dyq02270116@126.com (Y.D.); ssy2043@163.com (S.S.); 13739102719@163.com (J.M.); lxy5439@126.com (X.L.); 2 State Key Laboratory of Discovery and Utilization of Functional Components in Traditional Chinese Medicine, School of Pharmaceutical Sciences, Guizhou Medical University, Guian New District, Guiyang 550025, China; liushaobo8818@163.com; 3 The High Efficacy Application of Characteristic Natural Medicinal Resources Engineering Research Center of Guizhou Province, School of Pharmacy, Guizhou Medical University, Guiyang 550025, China; 4 Guizhou International Science & Technology Cooperation Base for Natural Drugability Research (Joint Laboratory), School of Pharmacy, Guizhou Medical University, Guiyang 550025, China

**Keywords:** acetyl tributyl citrate, icariin, infertility, network toxicology, network pharmacology, molecular docking, molecular dynamics simulation

## Abstract

Infertility is a prevalent clinical issue which disrupts normal human life and exerts an impact on fertility rates within the population. The increase in environmental pollutants, including acetyl tributyl citrate (ATBC), has given rise to concerns regarding their potential toxicity in infertility-related disorders. Icariin exhibits therapeutic effects on infertility, yet its mechanism of action against plasticiser-induced reproductive disorders remains unclear. This study aims to elucidate the potential toxicological targets and molecular mechanisms of ATBC-induced infertility, as well as the therapeutic targets and mechanisms of icariin in treating ATBC-induced reproductive disorders, through network toxicology, molecular-docking techniques and molecular dynamics simulation. Utilising the component-target database SwissTargetPrediction, the Similarity Ensemble Approach, PharmMapper, the ChEMBL database, and disease databases including the Therapeutic Target Database, OMIM, GeneCards, and DrugBank, 63 targets for ATBC-induced infertility and 33 targets for icariin treatment were identified. Screening via the STRING platform and Cytoscape 3.10.1 software yielded four core targets for ATBC-induced infertility—HSP90AA1, PIK3CA, CASP3, HRAS—and four core targets for icariin treatment—IL6, TNF, STAT3, and INS. Gene ontology (GO) and Kyoto Encyclopedia of Genes and Genomes (KEGG) pathway analyses revealed that ATBC-induced infertility correlates with pathways including pathways in cancer, prostate cancer, and PI3K-Akt signalling pathways. Conversely, the core targets of icariin therapy for related reproductive disorders are closely associated with tumour-associated signalling pathways and the AGE-RAGE signalling pathway. Molecular-docking and molecular dynamics simulation further confirmed the strong binding interactions between ATBC and infertility-related targets, as well as between icariin and core targets for treating reproductive disorders. This provides a theoretical foundation for understanding ATBC’s toxicological targets and the complex molecular mechanisms underpinning icariin’s treatment of infertility. It informs the development of strategies for icariin to prevent and treat infertility caused by exposure to ATBC-containing plastics or excessive ATBC contact.

## 1. Introduction

Reproductive health stands as a focal point in contemporary life sciences and medical research, constituting a pivotal theme within the realm of population and health. However, with the accelerating pace of global urbanisation and industrialisation, coupled with shifts in lifestyle patterns, the incidence of infertility has risen significantly in recent years, reaching rates as high as 15–20% worldwide [1]. The environment stands as a significant factor influencing infertility. The core conclusion is that prolonged exposure to harmful environmental factors can disrupt reproductive system function and reduce the probability of conception [2].

**Figure 1:**
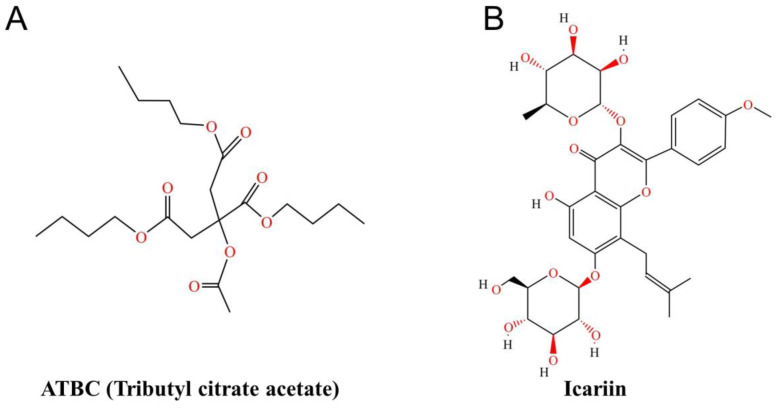
(**A**) Structural diagram of ATBC. (**B**) Structural diagram of icariin.

Plasticisers rank among the most crucial additives for rubber and plastics. These compounds also serve as raw materials and intermediates in numerous chemical products, finding extensive application across consumer goods and medical devices. Following the prohibition of several phthalate plasticisers due to their adverse effects, various emerging/alternative plasticisers have entered the market [3]. Acetyl tributyl citrate (ATBC) is a novel environmentally friendly plasticiser, whose structural formula is shown in Figure 1A. Derived from organic compounds extracted from multiple plant sources and esterified under various catalysts, it offers advantages over traditional plasticisers including low volatility, excellent cold resistance, and superior surface properties. ATBC is currently widely regarded as non-toxic, typically only considered hazardous at extremely high concentrations [4]. However, owing to its widespread use in food packaging and its physical rather than chemical bonding mechanism with polymers in plastics, ATBC readily leaches from packaging and other plastic products. It subsequently disperses into blood, bodily fluids, air, dust, environmental soil, and water, facilitating human exposure. Consequently, concerns regarding its human safety have grown significantly. Studies have shown that ATBC induces follicular DNA fragmentation, significantly increases apoptosis levels and impairs follicular viability. ATBC also targets the ovarian follicular pool and significantly reduces the number of primordial follicles, primary follicles, and secondary follicles, resulting in a clear impairment of ovarian function, suggesting that even low doses of ATBC may still have adverse effects on female reproduction [5,6,7]. Research by Japanese scholars has confirmed that plasticisers containing phthalates may interfere with the function of the endocrine system in vertebrates; one of them (ATBC) is able to disrupt the activity of reproduction-related hormones in fish, thereby impairing their normal reproductive processes [8]. In summary, acetyl tributyl citrate (ATBC) has been demonstrated to exert clear toxic effects on the reproductive system, leading to infertility.

Icariin is the primary flavonoid active component in the herbal medicine Epimedium; its structural formula is shown in Figure 1B. Modern research indicates that icariin exhibits androgen-like effects. Icariin can be employed to treat mammalian reproductive disorders by promoting testosterone synthesis and spermatogenesis, enhancing penile erection, and regulating reproductive function [9,10]. It also modulates the expression of reproductive hormone receptors and exerts beneficial effects on ovarian and uterine tissue structures [11]. Although research on ICA in relation to infertility has made some progress and identified certain mechanisms of action, the protective mechanism of ICA against infertility caused by routine exposure to plasticisers in daily life remains unclear.

Network toxicology is an interdisciplinary approach integrating bioinformatics, systems biology and cheminformatics. It provides a comprehensive framework for understanding how chemicals disrupt biomolecular networks and impair cellular function, potentially leading to disease by exploiting multi-omics data from genomics, proteomics and metabolomics studies [12]. Conversely, molecular docking simulates the intricate binding patterns of plasticisers with protein targets at the atomic level, elucidating potential mechanistic pathways through which these chemicals may contribute to carcinogenesis. Network toxicology offers rapid, convenient, comprehensive, and holistic perspectives when applied to dissecting molecular mechanisms of toxicity.

By integrating these advanced methodologies, this study endeavours to elucidate the molecular basis and target mechanisms underlying the development and progression of infertility resulting from routine exposure to the plasticiser ATBC. Building upon this, network pharmacology elucidates the connections between icariin and these targets. This study represents the first association of network toxicology with network pharmacology, aiming not only to provide novel insights for the safety assessment of plasticisers but also to contribute to the development of prevention and treatment strategies for infertility. It harnesses the potential of combining different approaches to enhance therapeutic efficacy within synergistic co-administration strategies.

## 2. Results

### 2.1. Toxicological Study of ATBC-Induced Infertility Based on Network Pharmacology

#### 2.1.1. ATBC-Predicted Targets and Infertility Targets

**Figure 2:**
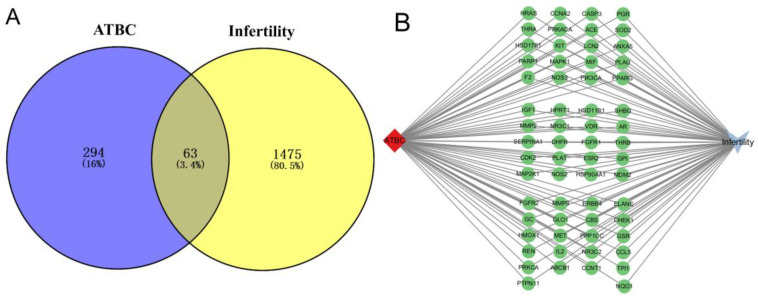
Intersecting targets of (**A**) TBC-induced infertility. (**B**) Component–target–disease visualisation network diagram.

ATBC targets were predicted by the above four databases, and a total of 364 targets were obtained after aggregation; 357 targets were finally obtained by de-weighting the aggregated targets (Table S1) and a total of 1542 infertility targets (Table S2) were obtained after aggregation and de-weighting of the data related to the four diseases, after which the intersection of ATBC-predicted targets and infertility targets was taken. A total of 63 targets were obtained, which were considered as the toxic targets of ATBC-induced infertility, as shown in Figure 2A.

#### 2.1.2. Establishment of Toxic Component–Target–Disease Network

In order to systematically analyse the molecular regulation mechanism of infertility induced by acetyl tributyl citrate (ATBC), the present study first carried out a systematic and integrated screening and validation of the potential targets of ATBC, and then identified 63 core targets that are closely related to the reproductive toxicity of ATBC. Taking ATBC as the core research starting point and combining the pathophysiological associations between these 63 targets and infertility diseases, we constructed and mapped the molecular interactions network of the ‘ATBC (exogenous pollutant component)-targets-infertility (disease)’ with the help of Cytoscape 3.10.1 visualisation software. As shown in Figure 2B, the network diagram clearly presents the direct binding relationship between ATBC and the 63 core targets, the functional association between targets and infertility diseases, and the synergistic regulatory network among targets, which reveals that ATBC does not work through a single target or pathway but through the modulation of multi-targets and multi-pathways. The component–target–disease network diagram constructed in this study provides a visual theoretical framework for the in-depth elucidation of the molecular regulatory network of ATBC reproductive toxicity, as well as an important target support and data basis for the subsequent targeted screening of active ingredients in traditional Chinese medicine, and the development of reproductive toxicity-protection strategies.

#### 2.1.3. PPI Network Establishment of ATBC-Induced Infertility Targets

**Figure 3:**
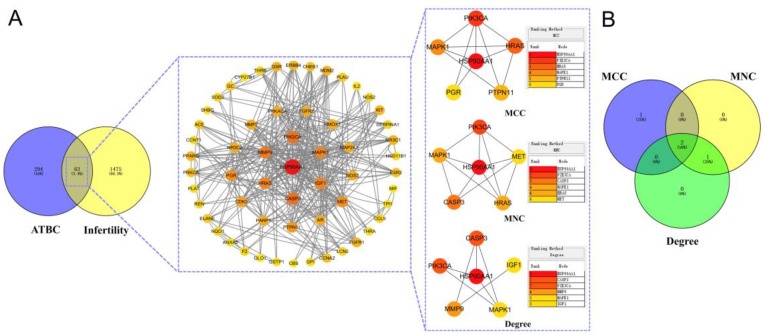
PPI network of ATBC-induced infertility targets and screening of its core targets. (**A**). Three algorithms for PPI networks and CytoHubba plugins. (**B**). Wayne diagram of the intersection of the results of the three algorithms.

The intersecting targets were imported into the online database String for analysis, and the protein–protein interaction (PPI) network diagram was obtained; the protein interactions file was imported into Cytoscape 3.10.1, the node size was adjusted according to the degree value for the visual optimisation, and the PPI relationship diagram was formed (Figure 3A and Table 1). The obtained data were plotted according to degree from large to small, the unrelated targets were deleted, the size of the targets and the colour depth were positively correlated, and the three algorithms NCC, NMC, and Degree of CytoHubba plugin in Cytoscape 3.10.1 were used to analyse the data. We identified the top three overlapping targets from the three algorithms as the final targets for ATBC-induced infertility and plotted a Venn diagram (Figure 3B). As shown in the PPI network, 4 core targets—HSP90AA1, PIK3CA, CASP3 and HRAS—were finally obtained.

#### 2.1.4. GO Analysis and KEGG Pathway Enrichment Analysis of ATBC Induced Infertility Targets

**Figure 4:**
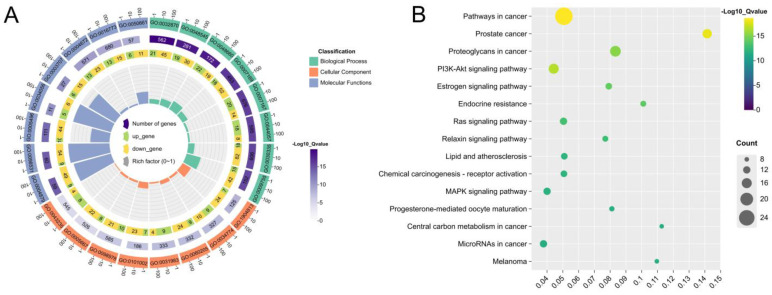
(**A**). GO function enrichment analysis of ATBC-induced infertility. (**B**). KEGG pathway analysis of ATBC-induced infertility.

To elucidate the mechanism of action of ATBC-induced infertility, we performed gene ontology (GO) enrichment analysis and Kyoto Encyclopedia of Genes and Genomes (KEGG) pathway analysis with the help of Metoscape database enrichment analysis, with *p* < 0.01 as the statistical significance criterion. The analysis results revealed that the GO enrichment analysis comprised three components: GO molecular function (MF), GO cellular component (CC), and GO biological process (BP). Where MF is mainly associated with nuclear receptor activity, ligand-modulated transcription factor activity, steroid binding, oestrogen response-element binding and other processes are closely linked, and CC is primarily associated with ficolin-1-rich granule lumen, secretory granule lumen, cytoplasmic vesicle lumen, vesicle lumen and other processes, BP collaboration with cellular response to hormone stimulus, response to steroid hormone, regulation of smooth muscle cell proliferation, and cell-surface receptor protein tyrosine kinase signalling pathway (Figure 4A). KEGG pathway enrichment analysis revealed 144 significantly enriched pathways (*p* < 0.05), mainly focusing on tumour regulation, signal transduction and other pathways. Among the pathways in cancer, prostate cancer and PI3K-Akt signalling pathways ranked high, as shown in Figure 4B.

### 2.2. Study of the Mechanism of Action of Icariin in the Treatment of Infertility Based on Cyberpharmacology

#### 2.2.1. Prediction of Icariin Targets and Infertility Targets

**Figure 5:**
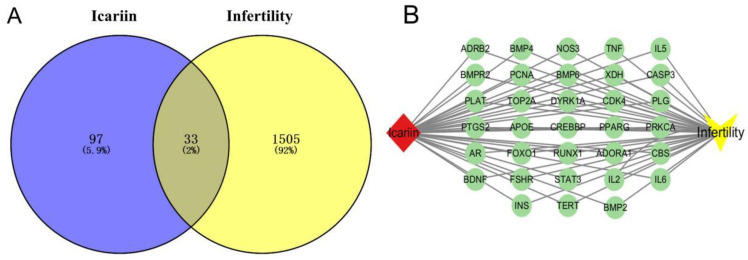
(**A**). Intersecting targets of ATBC-induced infertility. (**B**). Component–target–disease visualisation network map.

The above four databases predicted icariin targets; a total of 142 targets were obtained after aggregation, 130 targets were obtained after de-emphasis of the aggregated targets (Table S3), a total of 1542 targets were obtained after aggregation and de-emphasis of the data related to the four diseases, 1542 targets were obtained after aggregation and de-emphasis of the predicted targets and infertility targets, and 33 targets were obtained after intersection of the predicted targets and infertility targets of icariin, which were considered to be the targets of icariin. The intersection of Icarusin-predicted targets with infertility targets yielded a total of 33 targets, which were considered to be targets of Icarusin-treatment-induced infertility, as shown in Figure 5A.

#### 2.2.2. Establishment of Icariin–Target–Disease Network

In order to systematically analyse the molecular regulatory mechanism of icariin treatment for infertility, this study first conducted a systematic and integrated screening and validation of the potential targets of ATBC, and then identified 33 core targets that are closely related to the toxicity of icariin treatment for reproduction. Taking icariin treatment as the core research starting point and combining the pathophysiological associations between these 33 targets and infertility diseases, we constructed and mapped the molecular interactions network of icariin–target–infertility with the help of Cytoscape 3.10.1 visualisation software. As shown in Figure 5B, the network diagram clearly presented the direct binding relationship between icariin and the 33 core targets, the functional association between the targets and infertility diseases, and the synergistic regulatory network among the targets, which revealed that icariin does not work through a single target or pathway but through the regulation of multi-targets and multi-pathways. The component–target–disease network diagram constructed in this study provides a visual theoretical framework for the in-depth elucidation of the molecular regulatory network of icariin in the treatment of reproductive toxicity, and also provides important target support and data basis for the subsequent targeted screening of active ingredients of traditional Chinese medicines and the development of reproductive toxicity-protection strategies.

#### 2.2.3. PPI Network Establishment of Icariin Targets for Infertility Treatment

**Figure 6:**
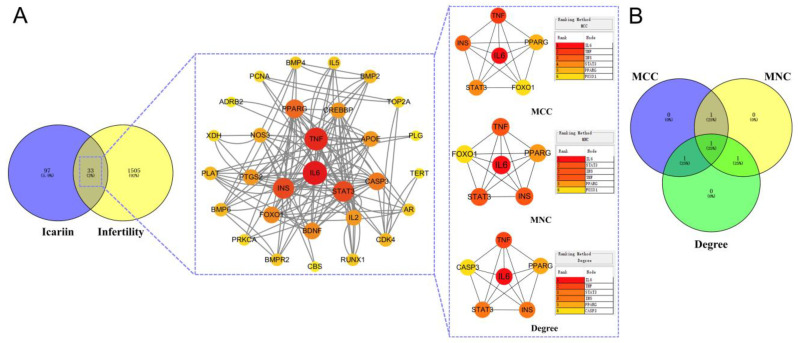
PPI network and screening of core targets for icariin treatment of infertility (**A**). Three algorithms for PPI networks and CytoHubba plugins. (**B**). Wayne diagram of the intersection of the results of the three algorithms.

The construction of the PPI network for icariin’s therapeutic targets in infertility and the screening of its core targets followed the methodology outlined in Section 2.1.3. The results are as follows: as shown in Figure 6 and Table 2, 4 core targets such as IL6, TNF, INS and STAT3 were finally obtained.

#### 2.2.4. GO Analysis and KEGG Pathway Analysis of Icariin Treatment Targets for Infertility

**Figure 7:**
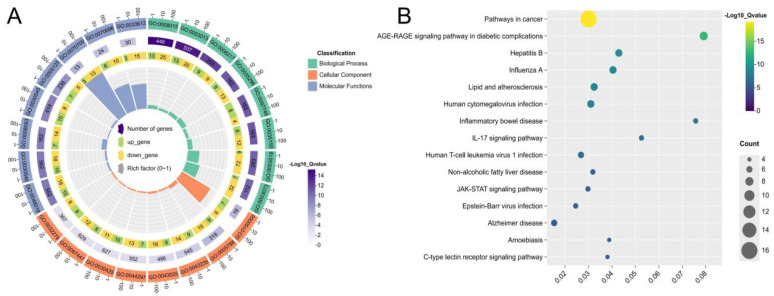
(**A**). GO function enrichment analysis of icariin for infertility. (**B**). KEGG pathway analysis of icariin for infertility.

In order to elucidate the mechanism of icariin in treating infertility, the enrichment analysis results from the Metscape database are as follows: MF is closely related to receptor ligand activity, signalling receptor activator activity, growth factor activity, and signalling receptor regulator activity; CC is closely related to fenzyme activator complex, endoplasmic reticulum lumen, receptor complex, and neuronal cell body; and BP is closely related to blood circulation, circulatory system process, regulation of blood pressure, and regulation of tube diameter (Figure 7A). KEGG pathway enrichment analysis revealed 89 significantly enriched pathways (*p* < 0.05) mainly focusing on tumour regulation, metabolic slow disease, neurodegeneration, signal transduction and other pathways; among the tumour-associated signalling pathways, the AGE-RAGE signalling pathway ranked the highest (Figure 7B).

**Figure 8:**
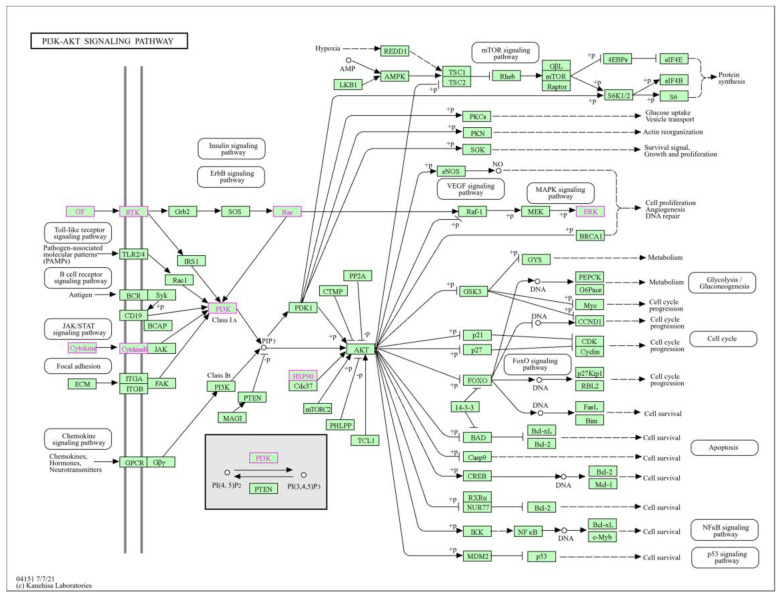
Core genes are enriched in PI3K-Akt signalling pathway. (Pink font is the core genes in this screen).

The screened targets of ATBC-induced infertility and the key targets of icariin for the treatment of its infertility were visualised by Pathview, and the core genes of the generation metabolic pathway—HSP90AA1, PIK3CA, CASP3, HRAS, IL6, TNF, INS, and STAT3—were mainly involved in the PI3K-Akt signalling pathway, whose ATBC induces infertility, and icariin treats its disease mainly by regulating this signalling pathway as shown in Figure 8. This study lastly focused on the PI3K-Akt signalling pathway, which is not only a core pathway closely related to infertility [13] but also a key pathway for icariin to ameliorate the toxicity of reproduction [14], which is highly consistent with the findings of the present study, and still needs to be further verified and developed through further experiments.

### 2.3. Analysis of Molecular Docking Results

**Figure 9:**
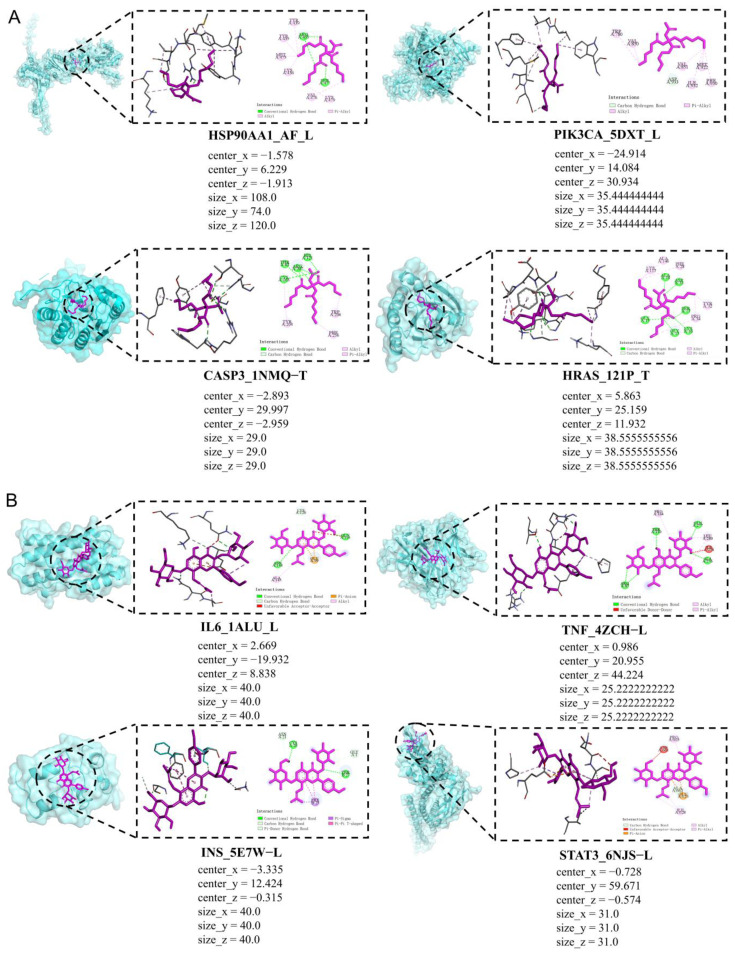
Display of molecular-docking results. (**A**). Molecular-docking plot of ATBC with core targets for induction of infertility. (**B**). Molecular-docking diagram of icariin with core targets for infertility treatment.

We performed molecular docking of the toxic component ATBC and its core target of infertility-causing targets, and icariin’s core target of infertility-treating targets, respectively, in which the molecular-docking map shows the results of plasticiser ATBC with the toxic targets of infertility, HSP90AA1, PIK3CA, CASP3, and HRAS, which are shown in Figure 9A.

For HSP90AA1, the docking energy of tributyl citrate acetate was −5.3 kcal/mol, with main interacting residues including TYR439, TYR435, MET475, LYS436, VAL476, LYS479, ARG405, and SER477, suggesting that hydrogen bonds and hydrophobic interactions stabilise the binding within the active pocket.

In the PIK3CA (PDB: 5DXT) complex, tributyl citrate acetate exhibited a binding affinity of −5.2 kcal/mol, interacting primarily with TRP780, VAL850, VAL851, MET922, PHE930, ILE932, and ASP933, which indicates that hydrophobic interactions dominate the stabilisation of the complex.

For CASP3 (PDB: 1NMQ), tributyl citrate acetate showed a docking affinity of −5.2 kcal/mol, interacting mainly with residues THR62, ARG207, HIS121, ASN163, TYR204, TRP206, and PHE256, where hydrogen bonding and hydrophobic interactions contributed to the stabilisation of the ligand–protein complex.

In the HRAS (PDB: 121P) system, the docking affinity of tributyl citrate acetate was −6.0 kcal/mol, and the ligand interacted with residues ALA146, LYS117, VAL18, PHE28, ASP33, SER17, GLY15, GLY13, CYS16, TYR32, and PRO34, indicating that hydrogen bonding and hydrophobic contacts play key roles in maintaining the binding conformation.

The results of molecular-docking plots of icariin with infertility toxicity targets IL6, TNF, INS, and STAT3 are shown in Figure 9B.

For IL6 (PDB: 1ALU), the ligand icariin demonstrated a stronger binding affinity of −6.7 kcal/mol, interacting with residues LYS120, GLN116, ASN144, GLU109, and ALA145, suggesting that hydrogen bonding and electrostatic interactions contribute to ligand stabilisation.

In the TNF (PDB: 4ZCH) complex, icariin showed a docking energy of −6.8 kcal/mol, interacting mainly with PRO164, THR210, ARG138, GLN177, LEU280, ILE278, and ALA276, indicating that hydrogen bonding and hydrophobic interactions collectively contribute to the stability of the ligand–protein complex.

In the INS (PDB: 5E7W) system, icariin showed a docking affinity of −7.3 kcal/mol, forming interactions with ASN21, CYS20, GLY1, THR27, and PHE25, which indicate that hydrogen bonding and π-related interactions help stabilise the ligand within the binding pocket.

For STAT3 (PDB: 6NJS), icariin displayed a binding affinity of −6.7 kcal/mol, with key interacting residues including PRO63, ASN60, ALA65, GLU65, and ILE628, suggesting the involvement of hydrogen bonding and hydrophobic contacts in maintaining the complex structure.

To verify the reliability of the docking protocol, a re-docking validation was performed using the native ligand binding sites. The root mean square deviation (RMSD) values between the two ligand conformations ranged from approximately 0.2194 to 0.4269 Å, which is significantly lower than the commonly accepted threshold of 2.0 Å, confirming that the docking method used in this study is reliable and capable of accurately reproducing the experimentally observed binding modes (Table 3).

### 2.4. Analysis of Molecular Dynamics Simulation Results

#### 2.4.1. Molecular Dynamics Simulation of ATBC with Toxic Targets

**Figure 10:**
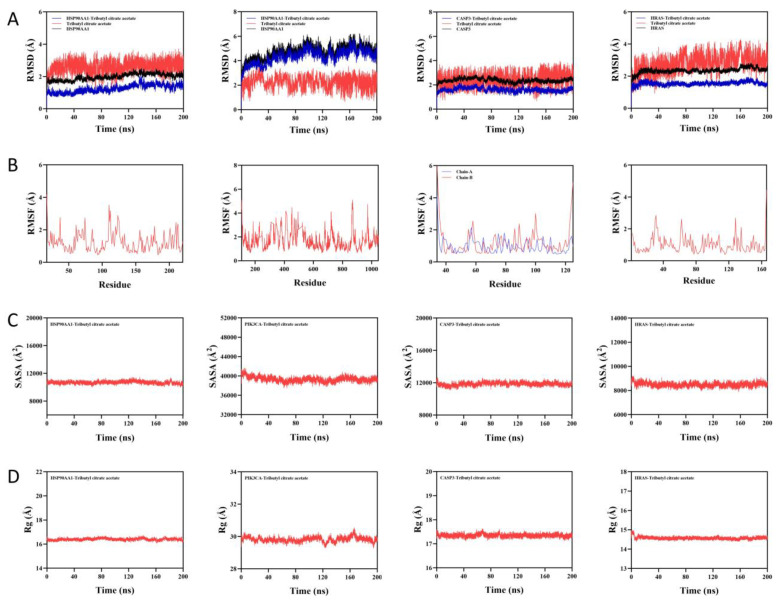
Molecular dynamics modelling of ATBC with toxic targets. (**A**). Time-dependent changes in the root mean square deviation of backbone atoms between the protein and ligand. (**B**). Analysis of root mean square fluctuations for each residue of the protein and ligand. (**C**). Time-dependent changes in the total solvent-accessible surface area of the protein and ligand. (**D**). Time-dependent changes in the radius of gyration of the protein and ligand. (Note: From left to right, HSP90AA1, PIK3CA, CASP3, and HRAS are listed).

The root mean square deviation (RMSD) is a good indicator of the conformational stability of proteins and ligands, as well as a measure of the extent to which the atomic positions deviate from their starting positions. The smaller the deviation, the better the conformational stability. Therefore, the equilibrium of the simulation system was evaluated using RMSD. As shown in Figure 10A, both the HSP90AA1 protein and HSP90AA1-ATBC complex system reached equilibrium after 40 ns and finally fluctuated up and down at 2.2 Å and 1.7 Å, respectively. The ATBC small molecule reached equilibrium after 10 ns, and finally fluctuated up and down at 2.8 Å. Both the PIK3CA protein and PIK3CA-ATBC complex system reached equilibrium after 180 ns. The PIK3CA protein and the PIK3CA-ATBC complex system both reached equilibrium after 180 ns and ultimately fluctuated up and down at 5.2 Å and 5 Å, respectively. The ATBC small molecules reached equilibrium after 50 ns, and ultimately fluctuated up and down at 3 Å. The CASP3 protein and the CASP3-ATBC complex system both reached equilibrium after 5 ns and ultimately fluctuated up and down at 2.5 Å and 1.7 Å, respectively. The ATBC small molecules reached equilibrium after 5 ns and ultimately fluctuated up and down at 3 Å. The HRAS protein and the HRAS-ATBC complex system both reached equilibrium after 5 ns, eventually fluctuating up and down at 2.5 Å and 1.6 Å, respectively. The ATBC small molecule reached equilibrium after 5 ns, eventually fluctuating up and down at 3.3 Å, respectively. Therefore, ATBC small molecules showed high stability when binding to HSP90AA1, PIK3CA, CASP3 and HRAS target proteins.

Root mean square fluctuation (RMSF) can indicate the flexibility size of amino acid residues in proteins. As shown in Figure 10B, the RMSF values of HSP90AA1-ATBC complexes were relatively low (mostly below 3.7 Å), the RMSF values of PIK3CA-ATBC complexes were relatively low (mostly below 5 Å), the RMSF values of CAsP3-ATBC complexes were relatively low (mostly below 3 Å), and the RMSF values of HRAS-ATBC complexes were relatively low (mostly below 3 Å). Thus, it is less flexible and more stable.

Solvent-accessible surface area (SASA) is a metric for assessing the surface area of proteins, and this simulation calculates the solvent-accessible surface area between the target protein and the small molecule. As shown in Figure 10C, the SASA of the complexes of HSP90AA1-ATBC, CASP3-ATBC, and HRAS-ATBC receptor bound to the ligands showed no significant changes, indicating that the binding of ligands has little effect on the protein structure. The PIK3CA-ATBC complex system showed slight fluctuations. It was demonstrated that binding of small molecules affects the binding microenvironment and leads to some degree of SASA changes.

Radius of gyration (Rg) can be used to describe the overall structural changes and can be used to characterise the tightness of the protein structure. As shown in Figure 10D, the HSP90AA1-ATBC, CASP3-ATBC and HRAS-ATBC complexes fluctuated more stably during exercise. It indicates that the small molecule–target protein complexes did not undergo obvious expansion and contraction during exercise, reflecting good conformational consistency and compact structure. The PIK3CA-ATBC complex system showed slight fluctuation during exercise, indicating that the small molecule–target protein complexes underwent conformational changes during exercise.

In conclusion, ATBC showed a stable binding mode with all the toxic targets, and the molecular dynamics simulation results further verified the stability and reliability of the binding between ATBC and its key toxic targets, confirming that the two could form a conformationally stable and tightly bound complex at the dynamic level, which provided a strong structural biological basis for the subsequent elucidation of its toxicity mechanism.

#### 2.4.2. Molecular Dynamics Simulation of Icariin and Therapeutic Targets

**Figure 11:**
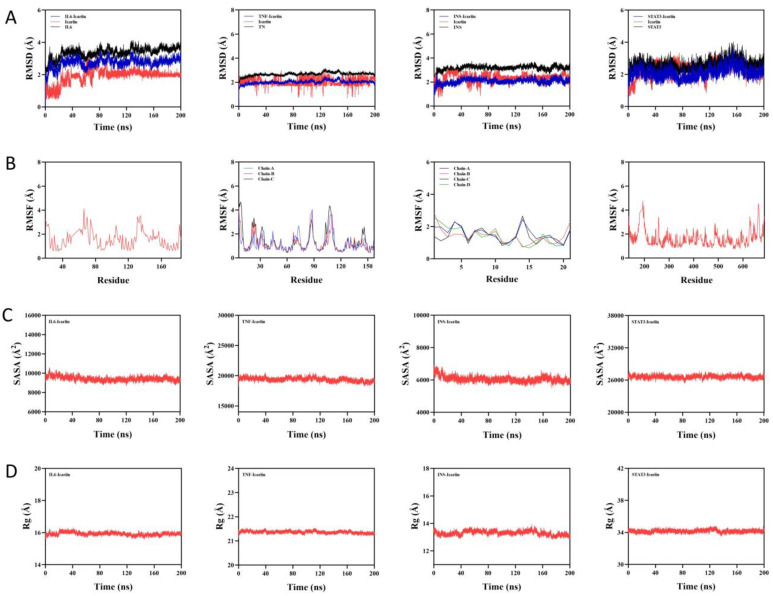
Molecular dynamics modelling of icariin with therapeutic target. (**A**). Time-dependent changes in the root mean square deviation of backbone atoms between the protein and ligand. (**B**). Analysis of root mean square fluctuations for each residue of the protein and ligand. (**C**). Time-dependent changes in the total solvent-accessible surface area of the protein and ligand. (**D**). Time-dependent changes in the radius of gyration of the protein and ligand. (Note: From left to right, IL6, TNF, INS, and STAT3 are listed).

As shown in Figure 11A, both the IL6 protein and IL6–icariin complex system reached equilibrium after 160 ns, and finally fluctuated around 3.7 Å and 3 Å, respectively; the small molecule of icariin reached equilibrium after 80 ns, and finally fluctuated around 2 Å. Both the system of TNF protein and TNF–icariin complex reached equilibrium after 80 ns, and finally fluctuated around 2 Å, respectively. The icariin complex system both reached simulated equilibrium after 150 ns, eventually fluctuating up and down at 2.6 Å and 2 Å, respectively. Icariin small molecules reached equilibrium after 150 ns, eventually fluctuating up and down at 2.4 Å. The INS protein and INS–icariin complex both fluctuated steadily between 40 and 150 ns, showing slight fluctuations in the later stages of the exercise, but always fluctuating below 3.3 Å and 2.2 Å, respectively. Icariin small molecules reached equilibrium after 90 ns and eventually fluctuated above and below 2.3 Å. Both the STAT3 protein and IL6–icariin complex systems reached equilibrium after 170 ns and eventually fluctuated above and below 2.9 Å and 2.4 Å, respectively. Icariin small molecules reached equilibrium after 170 ns and finally fluctuated around 2.7 Å. Therefore, icariin small molecules showed high stability when binding to IL6, TNF, INS and STAT3 target proteins.

As shown in Figure 11B, IL6–icariin complexes have relatively low RMSF values (mostly below 4 Å), TNF–icariin complexes have relatively low RMSF values (mostly below 4 Å), INS–icariin complexes have relatively low RMSF values (mostly below 3 Å), and STAT3–icariin complexes have RMSF values that are relatively low (mostly below 4 Å). Thus, it is less flexible and more stable.

As shown in Figure 11C, the SASA of the complexes of IL6–icariin, TNF–icariin, INS–icariin, and STAT3–icariin receptors did not change significantly upon binding of the ligands, suggesting that the binding of the ligands has less effect on the protein structure.

As shown in Figure 11D, IL6–icariin, TNF–icariin, INS–icariin and STAT3–icariin complexes fluctuated more stably during exercise. It indicates that the small molecule–target protein complexes did not undergo significant expansion and contraction during exercise, reflecting good conformational consistency and compact structure.

In conclusion, icariin can form a stable binding conformation with all the therapeutic targets, and the molecular dynamics simulation results further confirmed that icariin is firmly bound to the key targets and has a robust conformation, which provides important structural biological support for the in-depth understanding of its pharmacological mechanism of action.

## 3. Discussion

HSP90AA1 (heat shock protein 90 alpha family class A member 1) encodes heat shock protein 90 alpha (Hsp90α), a highly conserved inducible molecular chaperone of the heat shock protein 90 family, which plays a central role in cellular protein homeostasis regulation. HSP90AA1 is an essential molecular chaperone in male mouse spermatogenesis, and its deletion leads to a specific block in meiotic prophase I, triggering complete male sterility. This gene ensures the normal dissociation of post-coelomic processes and the association complex by maintaining the stability and activity of core meiotic factors such as Hsp70-2, NASP and Cdc2. This finding provides key animal experimental evidence for understanding the genetic mechanism of human idiopathic male infertility [15]. It has been shown that the rs11547523 locus of the HSP90AA1 gene is a risk factor for idiopathic male infertility in China, and it may affect sperm function by regulating the expression of this gene, which provides a new candidate target for genetic screening and diagnosis of male infertility, it is associated with HSP90AA1, a finding that could contribute to the development of diagnostics for male infertility [16]. In addition to its effects on men, HSP90AA1 can also cause infertility in women; its normal function is necessary for maturation of bovine oocytes and early embryo development, and inhibition of its expression or activity can trigger reproductive abnormalities by disrupting meiotic progression and embryo survival homeostasis; it has been hypothesised that the HSP90AA1 function abnormality may be one of the important factors leading to female infertility or embryo development failure [17]. In conclusion, HSP90AA1 is an important gene for infertility.

CASP3, known as Cysteine-dependent aspartate-directed protease 3 (CASP3), is a core molecule in the regulation of programmed cell death (apoptosis) in living organisms, and belongs to the caspase family, which plays a key role in the maintenance of cellular homeostasis, developmental regulation, and disease occurrence. It has been shown that CASP3 is a key regulatory molecule in male infertility by regulating the apoptotic homeostasis of spermatogenic cells and sperm function. Its physiological level of activity is necessary to maintain normal reproductive function, while an imbalance in activity directly triggers infertility phenotypes such as oligozoospermia, weak spermatozoa, and deformed spermatozoa [18]. It has been shown that CASP3 is the central executive molecule of testosterone-induced apoptosis in spermatogenic cells in the testis, and its mediated cascade of apoptotic responses is the key pathological mechanism of testosterone-deficient male infertility. Targeted regulation of CASP3 activity is expected to be a potential strategy for improving spermatogenic function and treating male infertility in testosterone-deficient patients [19], so, relatively, ATBC affects male sperm quality and testosterone levels by acting on CASP3 targets, leading to infertility.

HRAS (Harvey Rat sarcoma viral oncogene homologue) gene encodes H-Ras protein, which is an important member of the Ras family of small GTPases, and serves as a molecular switch to regulate cell proliferation, differentiation, and survival signalling pathways; its mutation has been closely associated with tumourigenesis and a variety of hereditary diseases (RASopathies). In recent years, it has been found to play a key role in the development and maintenance of the reproductive system and is significantly associated with the pathogenesis of infertility by the National Centre for Biotechnology Information. HRAS is classified as one of the core genes in the epigenetic regulation of spermatogenesis, and the abnormalities in DNA methylation and histone modification patterns have been focused on to clarify the role of this gene in normal spermatogenesis. The role of this gene in normal spermatogenesis and the pathological state of male infertility have been elucidated, and it is a key target linking epigenetic disorders and germ cell dysfunction. Abnormal epigenetic modification of HRAS is one of the important molecular mechanisms of male infertility, and its promoter, methylation level, can be used as a potential epigenetic marker for the diagnosis of male infertility [20].

In summary, ATBC may cause infertility by acting on the above toxicity targets, and the discovery of the pathogenic mechanism of plasticisers can clarify the core mechanism of plasticisers interfering with the reproductive system, such as targeting damage to gametogenesis, sex hormone receptor regulation, and embryo implantation-related pathways, and decipher the pathway of their insidious reproductive toxicity. Secondly, it will provide a basis for risk assessment and control and help define safe exposure limits for plasticisers in food packaging, mother and baby products, etc., so as to reduce the reproductive health risks of the population. Lastly, it will empower the research and development of low-toxicity alternatives and clinical interventions, guide the design of targeted protective drugs, and provide scientific direction for the repair of reproductive function in exposed populations.

IL-6 and TNF-α are common inflammatory factors in disease and have been shown to mediate the key pro-inflammatory cytokines in endometriosis-associated infertility, and both form an inflammatory network through the autocrine/paracrine pathway that interferes with the reproductive process from the three core aspects of sperm function, embryo development, and endometrial tolerance [21]. Studies have confirmed that IL-6 needs to form a complex with a soluble IL-6 receptor (sIL-6R), rather than acting alone, and interferes with sperm motility by targeting sperm-surface receptors, which is a key factor mediating gamete dysfunction in endometriosis-associated males, and ultimately reduces the efficiency of fertilisation, leading to infertility; the expression of the IL-6 receptor in spermatozoa of infertile males confirms that spermatozoa are the direct targets of IL-6, which provides a morphological and molecular basis for the involvement of IL-6 in the regulation of male reproductive function. IL-6 can activate downstream signalling pathways by binding to IL-6R on the surface of spermatozoa, affecting sperm viability, acrosomal reaction, spermatogonia, and other key physiological processes, and the abnormalities of the above indexes will directly reduce the ability of sperm to be fertilised, which will in turn lead to infertility. Chronic inflammation of the reproductive system is a common cause of male infertility, the secretion level of IL-6 rises under inflammation, and excessive IL-6 may exacerbate the oxidative stress damage of spermatozoa and promote sperm apoptosis by binding with spermatozoa IL-6R, which further deteriorates the quality of spermatozoa [22].

Some studies have explored the role of epithelial–mesenchymal transition (EMT) signalling around endometriosis in the diagnosis of infertility and in vitro fertilisation (IVF) outcomes, in which tumour necrosis factor (TNF), a core pro-inflammatory cytokine, is involved in infertility development and influences the outcome of IVF treatment by regulating the EMT process, and TNF contributes to the development of endometriosis by mediating EMT. TNF also reduces endometrial tolerance and hinders embryo implantation through regulated EMT abnormalities [23].

Insulin (INS) and its receptor (IR) signalling pathway are key hubs linking metabolic and reproductive functions, and their abnormality (insulin resistance/hyperinsulinaemia or insulin secretion) is widely involved in the pathophysiological process of infertility in both men and women by affecting the hypothalamo–pituitary–gonadal axis, ovarian/testis function, gamete quality, and endometrial tolerance, and plays a central role in infertility associated with polycystic ovary syndrome (PCOS) [24]. INS acts directly on hypothalamic gonadotropin-releasing hormone (GnRH) neurons, regulating their pulsatile secretion, and indirectly on gonadotropins (follicle-stimulating hormone (FSH) and luteinising hormone (LH)) by affecting their synthesis and release. This in turn leads to ovulation disorders (in women) or insufficient testosterone synthesis (in men), leading to infertility [25]. INS is involved in the regulation of testosterone synthesis in testicular mesenchymal cells and in supporting cellular functions, and testosterone is a key hormone for spermatogenesis. Insulin resistance leads to disturbances in the INS signalling pathway in the testis and a decrease in testosterone secretion, and it also affects the nutritional effects of support cells on spermatogenesis, resulting in oligozoospermia, hypospermia or spermatid malformations, and a decrease in fertilisation capacity [26].

STAT3 regulates uterine epithelial–mesenchymal interactions, endometrial metaplasia, placenta formation and ovarian follicular development by mediating cytokine (e.g., LIF, IL-6, IL-11) signals in the reproductive system, and its aberrant activation or tissue-specific deletion leads to failure of implantation, loss of pregnancy, and impairment of ovarian function. It is a key hub linking inflammation, immunity and reproductive function [27]. STAT3 production may alleviate symptoms of adenomyosis and endometriosis, improve endometrial tolerance, and potentially alleviate infertility without compromising the normal reproductive process [28].

In summary, in the process of screening ATBC pathogenic targets and icariin targets for infertility treatment, there is an intersecting target, CASP3, in its core targets, and the toxic component ATBC and the active ingredient icariin compete together to act on this target, wherein antagonists and agonists are two types of ligands acting on the same receptor target with completely opposite functions. Both are involved in mediating the inhibition and activation of physiological or pharmacological effects through the binding to and regulation of the receptor, so they play an antagonistic role in the infertility induced by the toxic component ATBC, and icariin plays an agonistic role in the therapeutic effect.

As we all know, although ATBC is an emerging plasticiser, some studies have shown that ATBC has a certain degree of toxicity even in long-term low-dose exposure, and its serum ATBC metabolites correlate significantly with the risk of breast cancer, suggesting that ATBC exposure may increase the risk of malignant transformation of female mammary epithelial cells by interfering with hormone metabolism or epigenetic regulation [29], and that ATBC has the potential to induce the risk of oral cancer; some scholars have elaborated its mechanism of action through bioinformatics [30,31] Meanwhile, the induction of CYP3A by ATBC and its regulation of hormone metabolism through the steroid/xenobiotic substance receptor (SXR) are well known to be closely related to the occurrence of hormone-related tumours (e.g., breast and prostate cancers), thus providing a metabolomic basis for the indirect involvement of ATBC in the risk of cancers. Taken together, the above findings indicate that ATBC not only has carcinogenic effects but also induces reproductive toxicity and leads to infertility, suggesting that its reproductive toxicity mechanism is closely related to multiple pathways of cancer development.

Moreover, experimental studies and bioinformatic predictions of ATBC-induced infertility have shown that in vitro ATBC can increase the area of TUNEL positivity, suggesting DNA fragmentation may be involved in apoptotic pathway activation, thus regulating apoptosis genes to induce infertility [5,6,7], and fish animal models have shown that ATBC modulates the expression of the genes chgH and vtg which are oestrogen-responsive genes, thereby affecting the reproductive system [8].

Some studies have shown that icariin can competitively bind to RAGE, a key target of the AGE-RAGE pathway, to block the over-activation of the pathway, thereby inhibiting downstream oxidative stress, chronic inflammation and apoptosis, and at the same time synergistically activating protective signalling pathways, such as PI3K/AKT, to ameliorate the decline in reproductive function associated with diabetes mellitus under its targeted regulation, and ultimately alleviate oligoasthenozoospermia and restore fertility [14].

There are more studies on icariin treatment of infertility, in which it significantly up-regulated the genes related to biomass synthesis and antioxidants (StAR, LHR, SIRT1, HIF1α, Bcl2, PCNA, AMH, Cyp19a1, Hsd17b1) as well as the iron death protection factors, GPX4 and SLC7A11, and significantly down-regulated the genes related to pro-apoptotic and inflammatory pathways (Bax, NF-κB, IL-6/STAT, P53). NF-κB, IL-6/STAT, P53) [32].

This study relies on computer-aided drug design techniques such as network pharmacology, molecular docking, and molecular dynamics simulation to theoretically predict the potential targets of toxic components and preliminarily analyse the drug–target interaction patterns. Among them, molecular dynamics simulation is mainly used to provide preliminary structural references and qualitative dynamic stability information, rather than to draw strict quantitative conclusions or strong mechanistic claims. Due to limitations in research methods and technical conditions, this study is still in the stage of theoretical prediction and virtual verification, and the relevant targets, pathways of action, and molecular mechanisms have not been confirmed by cell or animal experiments. Therefore, further systematic validation of the results of this study through cell function experiments, animal models, and other in vitro and in vivo studies is needed to clarify the true targets and biological effects of toxic components. In summary, this study only provides a preliminary theoretical basis and research ideas for this field, which can serve as a reference for subsequent experimental verification.

## 4. Materials and Methods

### 4.1. Collection of ATBC and Icariin Targets

The structures of ‘acetyl tributyl citrate’ and ‘Icariin’ were searched in the PubChem database for the SMILES (Simplified molecular input line entry system) structure and sdf structure, and the structures of the two components were searched in the following four databases for target prediction: Swiss Target Prediction database (http://www.Swisstargetprediction.ch/) (accessed on 15 January 2026), PharmMapper database (https://www.lilab-ecust.cn/pharmmapper/) (accessed on 10 January 2026), Similarity Ensemble Approach (SEA) database (https://sea.bkslab.org) (accessed on 15 January 2026) and ChEMBL database (https://www.ebi.ac.uk/chembl/) (accessed on 15 January 2026). In the databases, the species was selected as ”Homo sapiens”; the Swiss Target Prediction database was selected as it had a probability greater than 0, and the ‘acetyl tributyl citrate’ and ‘Icariin’ predicted targets were pooled and duplication.

### 4.2. Selection of Targets Associated with Infertility

The DrugBank database (https://www.drugbank.ca/), in terms of disease prediction and through the study of the link between drug targets and diseases as well as the intervention effect of drugs on diseases, can assist in the prediction of the development trend of diseases under the intervention of drugs, and can also discover the potential therapeutic drug targets of diseases, so as to provide a basis for the formulation of drug treatment plans for diseases and prediction of the disease process. It can also identify potential drug targets for disease treatment, providing a basis for the formulation of drug treatment programmes and prediction of disease processes [33].

In the Therapeutic Target Database (https://db.idrblab.net/ttd/) (accessed on 9 March 2026), this site provides information on proteins, nucleic acids, and therapeutic diseases, as well as pathways and drugs that target these targets. In terms of disease prediction, it can help researchers explore potential therapeutic targets based on known target–disease associations, and then predict the direction of disease treatment [34]. 

The OMIM database (http://www.omim.org) is an online catalogue of human genes and genetic diseases. It focuses on the collection and organisation of information on genetic diseases, and records in detail the clinical symptoms, inheritance patterns and related genes of various genetic diseases. In terms of disease prediction, it can be used to determine the likelihood of a disease caused by a specific genetic mutation by searching for the relevant gene information, which provides an important reference for the prediction and diagnosis of hereditary diseases [35].

The GeneCards database (http://www.genecards.org/) is an integrated database of gene-related data from approximately 200 web sources, including genomics, transcriptomics, proteomics, genetics, and clinical data. The rich genetic data can be used to study gene–disease associations in the prediction of diseases [36].

Using ‘Infertility’ as the keyword in the above databases, we searched the above databases to obtain the genes related to the above diseases; the inclusion criteria for infertility targets using GeneCards databases were identified with gene score > 1 [37]. We combined the search results of the four databases and deleted the duplicate targets, and the resulting targets are the relevant targets of the diseases.

### 4.3. Cyber Toxicological Mechanisms of ATBC-Induced Infertility

#### 4.3.1. Retrieval of Shared ATBC–Infertility Targets

The ATBC-predicted gene targets and infertility targets were entered into the online analysis tool Venny 2.1 (https://bioinfogp.cnb.csic.es/tools/venny/index.html) (accessed on 9 March 2026) to identify the intersections to obtain the intersected targets and form a Venny diagram.

#### 4.3.2. Establishment of ATBC-Induced Infertility PPI Network and Screening of Core Targets

The STRING database (https://string-db.org/) is a database used to analyse protein–protein interactions, which can be used to further study the mechanism of ATBC-induced diseases [38]. We uploaded the intersecting targets of ATBC and infertility into the STRING database, limited the study species to ‘homo sapiens’, set the lowest interaction score to the highest confidence = 0.700, and hid the nodes that were not interconnected with each other. The lowest interaction score was set to the highest confidence = 0.700, the unrelated nodes were hidden, and the protein interactions were obtained. The data file was exported and imported into Cytoscape 3.10.1 software, and the parameters were set so that the node sizes and colours reflected the degree values, the edges’ thicknesses reflected the binding scores, and the PPI network diagrams were established. In the subsequent phase of the study, three distinct algorithms—maximal clique centrality (MCC), maximum neighbourhood component (MNC) and degree centrality metrics via the CytoHubba plugin—were employed to evaluate the significance scores of each node and to identify pivotal connected hub genes. The integration of cardinal topological parameters, encompassing degree centrality, betweenness centrality, and closeness centrality, was undertaken in order to identify the top 6 hub targets with the highest cumulative values as key targets. We chose the three indicators of CytoHubba plugin function in Cytoscape software to comprehensively screen the core targets, and de-emphasised the top-ranked targets in the three algorithms to ultimately screen out the core targets; this study also chose the three methods to conduct a comprehensive analysis, which is innovative to a certain extent, and the results obtained have a higher degree of credibility and accuracy.

#### 4.3.3. Functional Enrichment and Pathway Analysis of Targets

Intersecting targets of ATBC-induced infertility were imported into the Metascape database (https://www.metascape.org/gp/index.html) (accessed on 9 March 2026) for personalised analysis, and functional analyses were carried out on biological process (BP), cellular component (CC), and molecular function (MF), under the species setting ‘H. sapiens’. Under the condition that the species is ‘H. sapiens’, functional analyses were performed on biological process (BP), cellular component (CC) and molecular function (MF). Based on the results of the previous analyses, the top 15 entries were selected, and the bar charts of GO enrichment analyses were plotted with the help of the ChiPlot online website (https://www.chiplot.online/). The KOBAS online analysis tool was used to analyse the KEGG pathway enrichment analysis of key target proteins, and to systematically analyse the biological components, molecular functions and related metabolic pathways they are involved in. The top 20 most significant enrichment entries were screened based on statistical significance (*p*-value), and the analysis results were imported into the ChiPlot online website for data visualisation and display in the form of bubble plots, in which the size of the bubbles reflected the number of genes, and the shade of the colour represented the level of significance of the enrichment [39]. We corrected for multiple testing using the Benjamini–Hochberg (BH) method with a default enrichment threshold of *p* < 0.05 and FDR < 0.25.

The signalling pathways and biological processes involved in core target-mediated infertility were comprehensively investigated to elucidate and highlight the key mechanisms. Finally, GO and KEGG enrichment results were visualised to effectively interpret and present our findings.

### 4.4. Web-Based Pharmacological Mechanism Study of Icariin for Infertility Treatment

In this study, we explored the mechanism of action of icariin in the treatment of infertility based on network pharmacology, in which the core steps of icariin potential target prediction, screening of disease–drug intersecting targets, protein–protein interaction (PPI) network construction, and screening of core key targets, as well as GO function enrichment and KEGG analysis, were all referred to in the analytical methods in Section 4.3 of this study, so as to ensure the reliability and consistency of the study results. 

### 4.5. Molecular Docking

Molecular-docking studies were performed using AutoDock 1.5.7 Tools for receptor preparation. The receptor protein was pre-processed by removing all water molecules and adding polar hydrogen atoms, after which it was treated as a rigid structure and saved as the docking receptor file. Ligand molecules were energy-minimised using Chem3D to obtain stable conformations; subsequently, hydrogen atoms were added, rotatable bonds were defined using AutoDock Tools, and the ligands were saved in the appropriate docking format. The docking grid box was defined based on the active site of the receptor, with appropriate centre coordinates and dimensions to ensure sufficient coverage of the potential binding region, and the corresponding docking parameter files were generated. Molecular-docking calculations were carried out using AutoDock Vina to obtain binding conformations and corresponding binding energies. The docking results were visualised and analysed using PyMOL 2.2.0. and Discovery Studio [39,40].

### 4.6. Molecular Dynamics Simulation

Molecular dynamics simulations (MDS) play a key role as a computational tool in studying the stability and actions of docked molecular assemblies [41]. To simulate the interaction of compounds with their target proteins, 200 ns of molecular dynamics simulations (MDS) of the complexes were performed using Gromacs 2022.6 software. Charmm 36 was chosen as the protein force field, Gaff2 was chosen as the ligand force field, the TIP3P water model was chosen to add solvents to the protein–ligand system and to create a water box with a periodic boundary of 1.2 nm, and the particle grid Ewald (PME) and Verlet algorithms are used to deal with electrostatic interactions, respectively. Then, 100,000 steps of isothermal isovolumetric ensemble equilibrium and isothermal isobaric ensemble equilibrium were simulated with a coupling constant of 0.1 ps and a duration of 200 ps. Both van der Waals and Coulomb interactions are calculated using 1.0 nm cutoff values. Finally, the system was simulated using Gromacs 2022 at constant temperature (310 K) and constant pressure (1 bar) for a total of 200 ns [42,43].

## 5. Conclusions

The results of this study show that the integration of network toxicology and network pharmacology not only builds a theoretical framework for revealing the toxicity mechanism of common plasticisers in daily life, but also clearly elucidates the therapeutic mechanism of icariin, an active monomer of traditional Chinese medicine, against infertility caused by the plasticiser ATBC. The ATBC toxicity targets and icariin therapeutic targets obtained in this study not only provide a new entry point for the prevention and control of reproductive toxicity caused by ATBC exposure but also expand the prevention of infertility.

## Figures and Tables

**Table 1:** Information on the PPI target induced by ATBC in infertility.

	Target	Degree	Closeness Centrality	Betweenness Centrality	Topological Coefficient
1	HSP90AA1	44	0.5	0.268578025	0.175324675
2	PIK3CA	26	0.404255319	0.054168741	0.264423077
3	CASP3	26	0.49137931	0.166395993	0.194139194
4	MMP9	24	0.47107438	0.246631347	0.152083333
5	HRAS	22	0.398601399	0.009800833	0.289772727
6	MAPK1	22	0.425373134	0.047206128	0.262337662
7	IGF1	22	0.425373134	0.08042035	0.215151515
8	MET	20	0.459677419	0.067207439	0.25952381
9	PGR	20	0.401408451	0.03899054	0.257142857
10	PTPN11	18	0.360759494	0.003561609	0.356481481
11	CDK2	16	0.404255319	0.033835927	0.282258065
12	MAP2K1	14	0.401408451	0.008472133	0.3640553
13	FGFR2	14	0.375	0.006598905	0.331632653
14	MMP2	14	0.438461538	0.027000099	0.274725275
15	HMOX1	14	0.401408451	0.157868045	0.222857143
16	PRKACA	14	0.37012987	0.00560977	0.313364055
17	AR	14	0.387755102	0.009615028	0.313364055
18	PARP1	14	0.398601399	0.042394758	0.264550265
19	NR3C2	14	0.38	0.040112042	0.266009852
20	NOS3	14	0.4453125	0.091210086	0.202380952
21	KIT	12	0.38	0.004873762	0.388888889
22	PPARG	12	0.419117647	0.057626998	0.270833333
23	GSR	12	0.314917127	0.106918742	0.25
24	FGFR1	12	0.38	0.003047271	0.392473118
25	ERBB4	12	0.385135135	0.003795276	0.403225806
26	MDM2	12	0.401408451	0.011439897	0.327956989
27	NR3C1	12	0.360759494	0.014502775	0.327380952
28	ESR2	10	0.375	0.012481024	0.353333333
29	PRKCA	10	0.37254902	0.005126488	0.361538462
30	CHEK1	10	0.393103448	0.003796495	0.366666667
31	CCNA2	10	0.347560976	0.007949934	0.4
32	PLAU	8	0.351851852	0.003194539	0.455882353
33	ELANE	8	0.345454545	0.075441518	0.35
34	GC	8	0.257918552	0.046592672	0.34375
35	ACE	8	0.37012987	0.01819588	0.329545455
36	THRB	6	0.316666667	0.002246377	0.481481481
37	NOS2	6	0.401408451	0.013619269	0.387096774
38	NQO1	6	0.3	0.013459647	0.5
39	SOD2	6	0.335294118	0.020485953	0.422222222
40	SERPINA1	6	0.27804878	0.010620269	0.523809524
41	LCN2	6	0.343373494	0.002787188	0.511111111
42	F2	6	0.27804878	0.010620269	0.523809524
43	CCL5	6	0.335294118	0.064969716	0.333333333
44	REN	6	0.327586207	0.00191704	0.5
45	THRA	4	0.290816327	0	0.615384615
46	PLAT	4	0.323863636	0	0.666666667
47	HSD11B1	4	0.279411765	0	0.8125
48	GSTP1	4	0.241525424	0	0.75
49	TPI1	4	0.347560976	0.036534043	0.5
50	GPI	4	0.272727273	0.009857978	0.5
51	SHBG	4	0.318435754	0	0.571428571
52	CCNT1	4	0.290816327	0	0.8125
53	IL2	4	0.26146789	0.007158521	0.5
54	ANXA5	4	0.337278107	0	0.666666667
55	GLO1	2	0.240506329	0	0
56	CYP27B1	2	0.205776173	0	0
57	MIF	2	0.252212389	0	0
58	CBS	2	0.309782609	0	0

**Table 2:** Information on the PPI target for icariin treatment of infertility.

	Target	Degree	Closeness Centrality	Betweenness Centrality	Topological Coefficient
1	IL6	28	0.617021277	0.264504652	0.282857143
2	TNF	26	0.58	0.211877395	0.318181818
3	STAT3	22	0.537037037	0.105856596	0.355371901
4	INS	22	0.557692308	0.09303503	0.340909091
5	PPARG	18	0.483333333	0.047263273	0.416666667
6	CASP3	16	0.537037037	0.273357964	0.358695652
7	FOXO1	14	0.46031746	0.02409688	0.478571429
8	BDNF	14	0.5	0.0401341	0.414965986
9	IL2	12	0.446153846	0.020197044	0.482456141
10	PTGS2	12	0.491525424	0.049917898	0.444444444
11	CREBBP	12	0.402777778	0.015489874	0.464285714
12	APOE	12	0.453125	0.010673235	0.491666667
13	NOS3	10	0.420289855	0.085139573	0.457142857
14	CDK4	8	0.381578947	0.197044335	0.305555556
15	BMP6	8	0.420289855	0.075944171	0.359375
16	BMP2	8	0.402777778	0.058292282	0.366666667
17	PLAT	8	0.414285714	0.068965517	0.533333333
18	IL5	6	0.414285714	0	0.647058824
19	RUNX1	6	0.371794872	0.001477833	0.58974359
20	BMPR2	6	0.311827957	0	0.733333333
21	BMP4	6	0.311827957	0	0.733333333
22	AR	6	0.367088608	0	0.666666667
23	XDH	4	0.358024691	0.002463054	0.5625
24	PCNA	4	0.281553398	0	0.75
25	TOP2A	4	0.281553398	0	0.75
26	PRKCA	4	0.376623377	0.002052545	0.75
27	PLG	2	0.295918367	0	0
28	TERT	2	0.278846154	0	0
29	CBS	2	0.298969072	0	0
30	ADRB2	2	0.371794872	0	0

**Table 3:** Molecular-docking experiments and RMSD validation of ATBC and icariin.

Protein	Ligand	Target Affinity (kcal·mol^−1^)	Comparison (Pubchem Id)	RMSD
HSP90AA1	ATBC	−5.3		
PIK3CA_5DXT	ATBC	−5.2	58204997	0.4269
CASP3_1NMQ	ATBC	−5.2	447402	0.3592
HRAS_121P	ATBC	−6.0	135398731	0.2615
IL6_1ALU	Icariin	−6.7	444305	0.3792
TNF_4ZCH-	Icariin	−6.8	4468930	0.3827
INS_5E7W	Icariin	−7.3		
STAT3_6NJS	Icariin	−6.7	139600322	0.2194

## Data Availability

Data is contained within the article.

## Supplementary Materials

The following supporting information can be downloaded at https://www.mdpi.com/article/10.3390/ijms27062918/s1.
